# Complications of Uterine Fibroids and Their Management, Surgical Management of Fibroids, Laparoscopy and Hysteroscopy versus Hysterectomy, Haemorrhage, Adhesions, and Complications

**DOI:** 10.1155/2012/791248

**Published:** 2012-04-09

**Authors:** Liselotte Mettler, Thoralf Schollmeyer, Andrea Tinelli, Antonio Malvasi, Ibrahim Alkatout

**Affiliations:** Department of Obstetrics and Gynaecology, University Hospital Schleswig-Holstein, Campus Kiel, 24105 Kiel, Germany

## Abstract

A critical analysis of the surgical treatment of fibroids compares all available techniques of myomectomy. Different statistical analyses reveal the advantages of the laparoscopic and hysteroscopic approach. Complications can arise from the location of the fibroids. They range from intermittent bleedings to continuous bleedings over several weeks, from single pain episodes to severe pain, from dysuria and constipation to chronic bladder and bowel spasms. Very seldom does peritonitis occur. Infertility may result from continuous metro and menorrhagia. The difficulty of the laparoscopic and hysteroscopic myomectomy lies in achieving satisfactory haemostasis using the appropriate sutures. The hysteroscopic myomectomy requires an operative hysteroscope and a well-experienced gynaecologic surgeon.

## 1. Introduction

In 25–30% of females, fibroids are diagnosed. Although the pathogenesis is not completely understood, we do know that myomas are hormone dependent and are derived from individual myoma cells and not from a metastatic process. Myomas are the most common benign solid tumours of the female genital tract. Although often asymptomatic, they may cause menorrhagia, metrorrhagia, infertility, pain, pressure symptoms haemorrhage, and repeated abortions. Whereas open abdominal myomectomy results in limited morbidity, similar to that with hysterectomy, laparoscopic myomectomy has resulted in remarkable advantages for the patient in medical, social, and economic terms, with less postoperative pain and shorter recovery time. Semm and Mettler published their first paper on laparoscopy myomectomy in 1980 [1]. Today, all myomas can be enucleated by this technique. Conventional laparoscopic surgery is supplemented by robotic support and abdominal entry often modified to NOS (natural orifice surgery) and NOTES (natural orifice transluminal endoscopic surgery), also called single-port entry.

## 2. Material and Methods

**Surgical Procedure**

 

### 2.1. Conventional and Laparoscopic Myomectomy

According to the extent of the fibroid, the optic trocar is placed within the umbilicus or 10 cm higher up in the midline or at Palmer's point. Under vision 2-3 additional ports are placed in the lower abdomen. The fibroid resection technique varies depending on the position and size of the myoma.

**(1) Pedunculated Myomas**

The pedicle of the myoma is coagulated with bipolar forceps and cut with laparoscopic scissors or resected after placement of loops or staplers. Sutures are not always required.

**(2) Subserous and Intramural Myomas**

After injection of the fibroid wall (extracapsular) with a vasopressin derivative solution, an incision is made vertically or horizontally after haemostasis with bipolar/monopolar forceps, ultrasound, or thermofusion at the convex point of the uterus, at the site of the underlying myoma and away from the adnexa. The incision is extended until the pseudocapsule is reached; the myoma dissection is then performed strictly within the capsule plane using two pairs of grasping forceps. Continuous haemostasis is performed with the bipolar forceps (ultracision). After myoma enucleation, the uterus is sutured along a seromuscular plane (edge to edge) using one or two layers of separate Poly Dioxanol Suture (PDS) stitches with extra- or intracorporeal knots. The suture pedicles should be within the wound. Continuous suction and irrigation are performed to minimize adhesion formation.

The myomas are then extracted via the suprapubic route by morcellation with an electric morcellator, followed by a laparoscopic check and careful peritoneal cleansing and haemostasis.

**(3) Cervical Myomas**

These myomas can be easily reached and enucleated transvaginally; however, a combined vaginal laparoscopic excision is sometimes necessary.

**(4) Focal Adenomyosis**

In cases of dysmenorrhoea resulting from well-discernible adenomyotic lesions, careful enucleation or resection of these areas is advised. Hysteroscopic ultrasound assistance may guide the resection.

**(5) Submucous Myomas**

Submucous myomas located within the uterine cavity are classified according to their myometrial infiltration into 3 categories. The hysteroscopic resection of these fibroids is easy and is performed with the resectoscopic loop in a slicing manner with bipolar or monopolar current.

**(6) Hysteroscopy**

Although the first exploration of the uterine cavity dates back to Bozzini, the modern CO_2_ liquid, office, and operative hysteroscopy (resectoscopy) was developed by Hans Lindemann in Hamburg in 1972 and improved and modified by Bettocchi et al., Loffer et al., Cooper et al., Gallinat, and Campo et al. [2–8]. Recently, vaginoscopy has again been applied to guide the diagnostic hysteroscope from the vagina into the uterine cervix without any traction of a cervical tenaculum. With saline solution as the distension medium and under exact pressure and flow control, the visualization of the uterine cavity and its pathology are visible, including the synechiae, septum, endometrium, cervical canal, uterine cavity with uterine ostia, polyps, and fibroids. The most modern system offers continuous suction of resected material. The ESHRE classification of 1993 makes it easier to describe the location of the fibroid [5, 9].

### 2.2. Single-Port Myoma Enucleation

Myoma enucleation can easily be performed via all kinds of single-port entries (SILS: single incision laparoscopic surgery, LESS: laparo-endoscopic single-site surgery), natural orifice surgery (NOS), and natural orifice transluminal endoscopic surgery (NOTES). The problem, however, lies in the morcellation of the material and the extraction. The answer might be found in homogenization to powder.

### 2.3. Robotic Myomectomy

Once the learning curve is over, the da Vinci robot offers a more precise technique for every surgery, including myomectomies. Robotic suturing is easier and faster. In my opinion, one day all surgical procedures will be performed robotically.

After 30 years of experience in performing and teaching laparoscopic myomectomies, Professor Mettler has had the opportunity to perform a number of robotically assisted myomectomies with the da Vinci robot. The procedure is easily, can be, performed with less blood loss than at laparoscopy, and suturing can be carried out more precisely. However, three-dimensional vision and articulated instruments may also serve the same purpose. Robotic surgery is fascinating, but a financial revolution would have to occur for it to be accepted throughout the world, not only for cancer surgery but also for procedures such as myomectomy.

### 2.4. Laparotomic Myomectomy

A laparotomic myomectomy may be performed if the myoma is >20 cm in diameter, located at a very critical point, or suspected of being a sarcoma. More than 10 fibroids may require a laparotomy. The decision is taken by the surgeon.

### 2.5. Hysterectomy

**(1) Classic Intrafascial Supracervical Hysterectomy (CISH)**

In cases of adenomyosis or diffuse myomatosis of the uterus with no cervical pathology, CISH or LASH should be the method of choice for hysterectomy [10]. At CISH, the transformation zone of the cervix is cored out in addition to the subtotal uterine resection.

**(2) Laparoscopic Subtotal Hysterectomy (LASH)**

LASH has proved to be a safe, quick, and very atraumatic hysterectomy technique. The advantage of the LASH procedure is that it can be performed on nulliparous patients, patients who have not previously had a vaginal delivery, and patients who have had previous abdominal surgery. In these cases, the uterus is morcellated, but no colpotomy is performed.

**(3) Total Laparoscopic Hysterectomy (TLH)**

Indications for TLH include benign gynaecological alterations such as fibroids, endometriosis, and dysfunctional uterine bleeding in patients for whom vaginal surgery is contraindicated or cannot be performed. TLH may be performed for possible malignant indications such as early endometrial cancer, early localized small cervical cancers (trachelectomy), and also in the early stages of ovarian cancer with lymphadenectomies. The laparoscopic part consists of preparation of the uterus, the cervix, and complete dissection of the vaginal stump.

**(4) Vaginal Hysterectomy**

When Langenbeck first performed a vaginal hysterectomy in 1813, the discipline of gynaecology was founded. Since then vaginal access has been the privilege of the gynaecological surgeon. In 1939, according to the French surgical expert Doyen, no one could call himself a gynaecologist if he had not performed a vaginal hysterectomy [11].

Vaginal hysterectomy is still a central feature of gynaecological discussion. The gynaecologist only considers other access routes for the exploration of the minor pelvis if vaginal access cannot give a clear diagnosis and possibility of treatment. Today, however, it appears that only very skilled vaginal surgeons still operate on fibroids via the vaginal route using vaginal morcellation with a knife.

**(5) Abdominal Hysterectomy**

Abdominal hysterectomy today is a safe technique. There is no more fear of infection, thrombosis, or other morbidities. In the last 40 years of the 20th century, an explosive increase in the number of hysterectomies took place. Even in Germany, the method of choice for bleeding abnormalities, myomas, and other pathology was always laparotomy and hysterectomy. Large diffuse fibroids and multiple fibroids still sometimes require an abdominal hysterectomy.

### 2.6. Adhesion Prevention

Under the multitude of adhesion, prevention agents presented over the last 10 years, Adept (4% icodextrin solution) hyalobarriers and SprayShield (a polyethylene glycol sprayable liquid that polymerizes within seconds to a hydroabsorbable gel) appear to be the most promising substances. Hya-Corp endogel, an antiadhesion gel on hyaluronidase basis, is also highly recommended.

### 2.7. Haemorrhage

Haemorrhage can occur in submucous fibroids although seldom in intramural fibroids. The only therapy is myomectomy, no further discussion is necessary.

## 3. Results

### 3.1. Comparison of Removal of Single and Multiple Fibroids

From January 2002 till September 2007, 335 women, with a mean age of 35.2 years, underwent single or multiple intracapsular laparoscopic myomectomy in multiple gynaecological centers. Of these patients, 62 had undergone a previous caesarean section (18.7%). All patients signed an informed consent prior to inclusion in this study, as approved by the local institutional research ethics committee.

The selected patients requested a myomectomy for the following associated symptoms: pelvic pain, menorrhagia, and growth of myomatic nodules, as verified by ultrasound. A few requested myomectomy in view of a future pregnancy. Exclusion criteria for the investigation were as follows: previous uterine surgery (excluding caesarean section), presurgical treatment with GnRH analogs, a history of gynaecological malignancy, and primary subfertility.

Exclusion criteria for preoperative GnRH analog treatment were due to reported increased risk of recurrence, a possible delay in the diagnosis of leiomyosarcoma, a risk of massive hemorrhage from degeneration, a greater difficulty in finding the cleavage plane, and a greater extent of hyalinization phenomena [12, 13]. All fibroids were selected through standardized transvaginal ultrasound myoma mapping; all patients had subserous and/or intramural fibroids, and transvaginal ultrasound dates were recorded for postsurgical evaluation.

In the preliminary study setup, clinicians selected all women who had 3–6 myomas as patients to be submitted to multiple myomectomy. In the study setting, the size of the myoma before laparoscopy was between 4 and 9 cm. This limit was selected by surgeons to avoid longer operation times and pointless uterine trauma for smaller fibroids.

To give homogeneity to the intracapsular laparoscopic myomectomy, the authors excluded pedunculated, cervical, and intraligamentary myomas because they are extra myometrium.

Patients were first subdivided into two nonrandomized groups: 195 women with a single myoma in group I and 140 with two or more myomas (less than four) in group II.

The women underwent a standardized technique, described by Mais et al. in 1996, which was performed by the same well-trained residents in referred gynecological centers [14]. This standardized technique involved hysterotomy, pseudocapsule incision, extracting the myoma by stretching and uterine suturing.

Before incising the myometrium covering the myoma, a 10 cc solution of vasopressin or diluted adrenalin (1/100) was injected into the tissue layers, to make tissue ischemia easier and better delineate the cleavage plane and the pseudocapsule.

The laparoscopic intracapsular myomectomy was performed using the following intracapsular technique: the myometrium was incised vertically using a monopolar scalpel after identifying the plane between the pseudocapsule and the myoma.

The purpose of hysterotomy, with regard to the length and depth of the uterine incisions, was to expose the myoma pseudocapsule. Thus, the depth of the uterine incision was adapted to localize fibroids in the uterus and to show the surrounding structure. The length of the incision was, on average, based on the fibroid diameter. Generally, the length in each group was limited to the length of the myoma downside, and the depth of the uterine incision was limited to the depth of the pseudocapsule.

**Figure 1:**
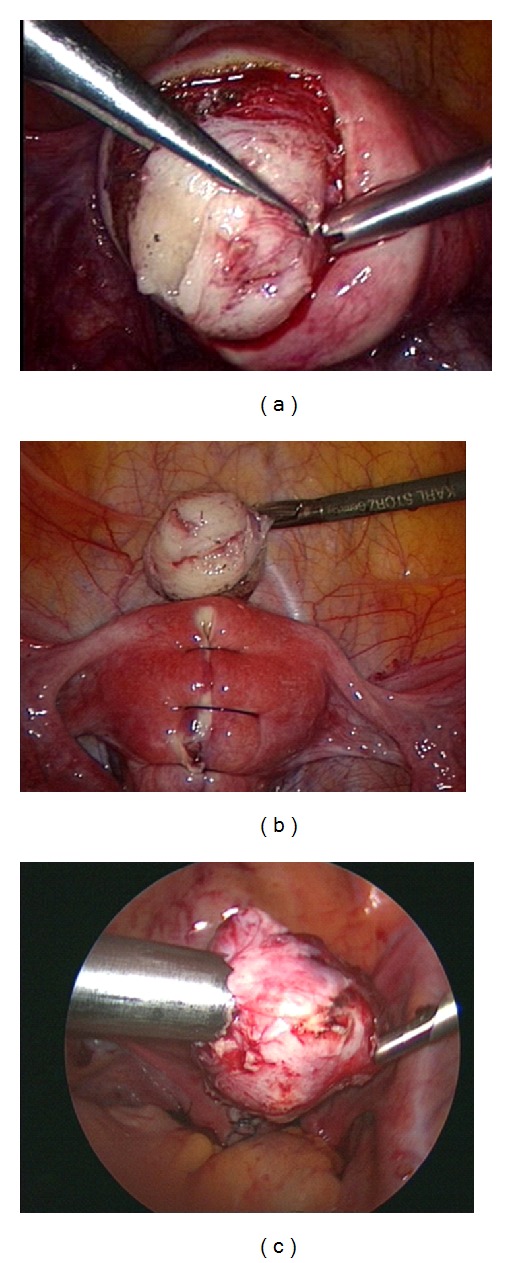
Myomectomy: (a) intraoperative sight of the myoma and its surrounding vascularized capsula. (b) Reconstruction of the uterine wall after excision of the tumor. (c) Removing the myoma by morcellation.

To dissect the pseudocapsule connective bridges from the surrounding myometrium and to allow the enucleation of the intracapsular myoma, the authors used a monopolar crochet needle or bipolar clamp-scissors (Gyrus PlasmaKinetic AMS) and myoma drills or Collins forceps (Figure 1).

In all procedures, myomas were removed using electrical morcellators (Karl Storz Endoscopy and Gynecare, Johnson & Johnson), and the myometrium was sutured using intra- and extracorporeal single or double stitches, with 0 absorbable poliglecaprone monofilament.

Surgeons approximated the clear edges of the uterine defect (linked to intracapsular method), with introflexing single U-stitches, at 1 cm increments. If the myometrial defect was deep or large, it was repaired by suturing with multiple intro-flexing single stitches, followed by serosal repair with multiple introflexing single stitches. Albeit difficult, at times the myometrium required two-layer suturing. Sutures were then applied at 1 cm increments, using extra- or intracorporeal adequate knot tying, depending on bleeding: extracorporeal for larger bleeding and intracorporeal for smaller.

A standard transvaginal ultrasonography detected problems before discharge ll procedures were based on the following surgical parameters: infiltration into the myometrium (number of patients per group), total operating time (in minutes), intrasurgical blood loss (in mL), postsurgical bleeding (mL in drainage), need for painkiller drugs (number of patients per group), postoperative fever (number of patients with fever >38°C after 24 hours and for the first 2 days of hospitalization), postoperative antibiotic administration (number of patients who requested therapy), duration of hospitalization (for 24 or 48 hours), and postoperative clinically significant intramyometrial hematoma >3 cm, as detected by a standard transvaginal ultrasonography before discharge [15].

The symptoms of patients submitted to laparoscopic myomectomy in group I were pelvic pain in 134 patients (68.7%), menorrhagia in 91 patients (46.6%), and growth of myomatic nodules, confirmed by ultrasound (US), in 119 (61.02%). In group II, the same symptoms were reported by 59 (42.1%), 77 (55%), and 73 patients (52.1%), respectively.

A comparison of participants' baseline characteristics did not show any statistically significant differences (*P* < 0.05) (Table 1).

The mean total laparoscopic operating time was  60 ± 7.2  minutes in group I and was significantly longer in group II with  97 ± 8.9  minutes (*P* < 0.05 in the Student's *t*-test analysis). No statistical differences (*P* > 0.05) were found in the amount of mean intraoperative blood loss (140 ± 4.7 mL in group I versus  175 ± 6.8  in group II), the number of catheters placed inside the pelvis for postsurgical drainage (78 women in group I versus 51 in group II), the need for painkillers (81 women in group I versus 56 in group II), postoperative fever on 1st hospitalization day (22 patients in group I versus 13 in group II), or postoperative antibiotic administration (16 women in group I versus 9 in group II). In the Student's *t*-test analysis, no statistical differences (*P* > 0.05) were found for the duration of hospitalization with respect to the number of women discharged in 24 hours (140 women in group I versus 99 in group II) and in 48 hours (55 patients in group I versus 41 in group II) or in the detection of postoperative intramyometrial hematoma (>3 cm) with ultrasound during the patients' hospitalization (13 women in group I versus 8 in group II).

No laparoconversion was performed in this study. Normally, all patients leave the operating theater with an analgesic mixture of ketorolac or tramadol in pump; however, if patients request more analgesic, nurses are accustomed to administer “on demand” 30 mg ketorolac i.v. or 100 mg tramadol i.v. in allergic patients (Table 2).

There were no significant differences in short-term complications, such as acute anaemia (with Hb < 9 g/dL, hematocrit (Hct) < 30 or red cells (RC) < 3,500,000 millions/mm^3^), postoperative excessive abdominal bleeding (more than 50 mL of blood in the catheter inside the pelvis for postsurgical drainage), and urinary tract infections (detected by positive urine culture after Foley removal) between the two groups (Table 3).

All women became pregnant spontaneously, without assisted fertility techniques or pharmacological preconception treatments for subfertility, and none of the remaining patients decided to receive additional assisted reproductive technology (ART). 

### 3.2. Spontaneous Pregnancy Rate after Laparoscopic Myomectomy in Kiel

In a series of 1032 laparoscopic myomectomies, there were only 6 complications (Kiel University Department of Obstetrics and Gynaecology, Germany). Out of 130 patients desiring pregnancies, 78 (60%) became pregnant. Among the 78 pregnancies, there were 6 abortions, 60 spontaneous deliveries, and 18 caesarean sections. Eight sets of twins and one set of triplets were reported [16].

### 3.3. Update on Laparoscopic Myomectomies and Hysteroscopic Treatment

**Figure 2:**
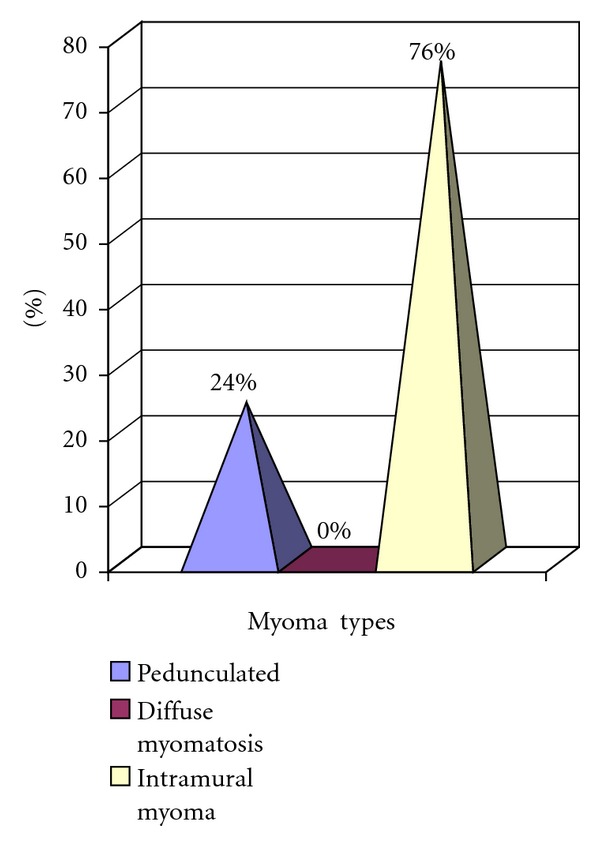
Types of myomas according to their type of surgery laparoscopic myomectomy (*n* = 178).

In laparoscopic myomectomy, the types of myoma treated were as follows: pedunculated-subserous 24%, intramural 76%, and diffuse myomatosis 0% (Figure 2). The average diameter of the myomas excised laparoscopically was 4 ± 2.1 cm (range 1–10 cm), with 57% having a diameter >4 cm. A drain to observe possible intraabdominal bleeding was placed in 168 laparoscopic myomectomy cases and removed after 24 h. No patient required a blood transfusion.

The mean operating time for laparoscopic myomectomy was 90 min (range 25–215 min). The mean length of hospitalization for laparoscopic myomectomy was  2 ± 0.5  days (range 2–6 days). In Germany at the time the study was carried out, the hospital was paid by insurance companies according to the number of days the patient was hospitalized and not according to the procedure performed. This resulted in longer hospital stays. This practice is now changing.

## 4. Patient Outcomes

### 4.1. Immediate Complications/Late Complications

Two patients had hematoma formation within the abdominal wall. In one patient, the epigastric artery was stitched immediately; in the second, patient a growing haematoma was found in the left abdominal wall four hours later. No late complications, such as bleeding, urinary tract infections, or bowel lesions, occurred.

### 4.2. Multiple Fibroids and Need for Subsequent Surgery

Six to eight weeks later, a second-look laparoscopy with myomectomy was suggested for seven of the 178 patients who had undergone laparoscopic myomectomy. The individual surgeon decided in each case to operate in two sessions. No conversions to laparotomy occurred. In our department, patients with larger fibroids undergo a mini-laparotomy/fibroidectomy directly. As seen in Figure 4, the majority of patients had only one myoma, either pedunculated-subserous or intramural (Figure 4).

### 4.3. Relief of Symptoms

To date, of the 178 patients, only two had further surgery for complaints associated with bleeding abnormalities and the need for hysterectomy.

## 5. Fertility Outcome

**Figure 3:**
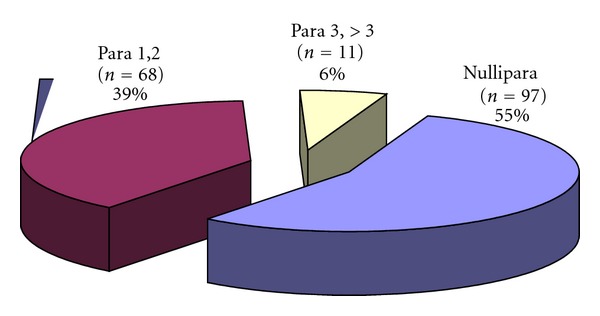
Incidence of myomas related to parity in laparoscopic myomectomies.

**Figure 4:**
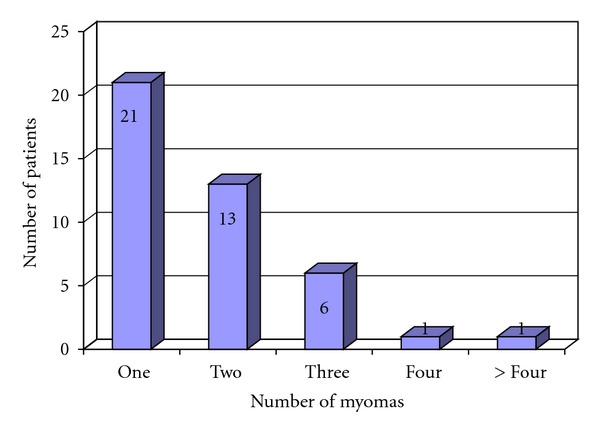
Number of pedunculated sub serous myomas (*n* = 42) in laparoscopic myomectomy.

The nulliparous women had the highest number of myomas, and the least were found in women who were para >3 (Figure 3). When the selection criteria were strictly adhered to, and there was no other associated infertility, laparoscopic myomectomy increased implantation rates [17, 18].

Over a period of 10 months to 4 years postlaparoscopic myomectomy, a 55% pregnancy rate was achieved. The caesarean section rate was 30%.

Laparoscopic myomectomy allows a safe vaginal delivery [19–21]. Other possible techniques are the embolization of uterine arteries or laparoscopic uterine artery ligation [22].

Patients who have undergone laparoscopic or laparotomic myomectomy and become pregnant should inform their gynecologists in case a caesarean section should be necessary.

## 6. Postoperative Resumption of Normal Life

Patients treated by laparoscopic myomectomy returned to work within 4–6 days after the initial surgery.

## 7. Discussion

### 7.1. Advantages of Laparoscopic Myomectomy over Open Myomectomy

In a literature search using Medline, 1400 citations were found. Selected papers were screened for further references. Criteria for selection of literature were the number of cases (excluded if less than 20), methods of analysis (statistical or non-statistical), operative procedure (only universally accepted procedures were selected), and the institution where the study was done (specialized institutions for laparoscopic surgery) [23]. Out of a total of 30 articles, only six met the selection criteria.

Chapron et al., in a 2002 meta-analysis of published data from randomized clinical trials, looked at risks facing patients after laparoscopic myomectomy (1809 patients underwent laparoscopy and 1802 patients laparotomy) [24]. They found that the overall risk of complications was significantly lower for patients operated by laparoscopy [24].

Holzer et al. are accredited with the first double-blind study in pain control after laparoscopic myomectomy [25]. After surgery, all the patients had similar dressings and therefore none of the patients knew who had undergone which type of surgery (19 had laparoscopy and 21 had laparotomy). The investigators were also kept in the dark. Analysis done on completion of the study showed that laparoscopic surgery had clear advantages over laparotomy as far as pain control is concerned [25].

Rossetti et al., in their review published in April 2001, looked at the rate of myoma recurrence following either laparoscopic or laparotomic myomectomy (162 patients, 82 for each type of surgery) [26]. Patient followup continued for up to 40 months. At the end of this time, 11 in the laparoscopy and 9 in the laparotomy group had suffered a recurrence. Analysis did not show any statistical significance [26].

To evaluate our own laparoscopic myomectomies, let us present our most recently evaluated series of 178 myomectomies in Kiel [16].

The following five criteria were evaluated in a questionnaire sent to all patients 10–24 months after surgical treatment: (1) immediate or late complications, (2) need for next, repeat or subsequent second-look surgery, (3) relief of symptoms, (4) fertility outcome, and (5) resumption of normal life.

#### 7.1.1. Signs and Symptoms of Myomas

Of the 178 patients, 24% had a pelvic mass with pressure symptoms, 17% metrorrhagia, 14% pelvic pain, and 40% infertility.

#### 7.1.2. Immediate Complications/Late Complications

Two patients had haematoma formation within the abdominal wall. In one patient, the epigastric artery was lacerated and stitched immediately; in the second patient a growing haematoma was found in the left abdominal wall four hours later. No late complications, such as bleeding, urinary tract infections, or bowel lesions, occurred. The incidence of myomas related to parity: 97 = 55% were nulliparous, 11 = 6% para 3, and > 3.68 = 39% para 1 and 2.

### 7.2. Complications and Conclusion

Complications can arise from the location of the fibroids. These complications range from intermittent bleedings to continuous bleeding over weeks, from single pain episodes to severe menorrhagia and chronic abdominal pain with intermittent spasms, from dysuria and constipation to chronic bladder and bowel spasms and even to peritonitis. Infertility may be the result of continuous metro and menorrhagia, leading to chronic infection and uterine spasms up to nonimplantation. Possible complications resulting from treatment of these disorders are haemorrhages, infection, adhesions, and secondary pain resulting from the treatment efforts.

Uterine myomas are very common in women of reproductive age and their diagnosis does not always require surgery. With the introduction of GnRH analogs medical treatment has become feasible. This medical treatment is capable of inducing a substantial reduction in the volume of the myoma (up to 50%) by reducing circulating oestrogen levels. Maximum reduction is achieved by a twelve-week therapy. However, after cessation of therapy, the myoma can again increase its size, up to the initial diameter, within three months. Myomas may be responsible for metrorrhagia, pelvic pain, anaemia, infertility, and abortion. When these symptoms occur, primary treatment might be analgesics for pelvic pain, iron for anaemia, or even IVF for infertility. However, if this treatment is unsuccessful, surgery is always indicated [27–29].

When surgery is indicated in cases of myomas, laparoscopic surgery is the primary choice. Depending on the alternatives available to the surgical team, the endoscopic treatment may be conventional laparoscopic, robotic, resectoscopic, using single or multiple ports with NOS or NOTES or hysteroscopic, according to the location.

Preoperative assessment is important to determine the operative strategy according to size, number, and location of the myomas. Precise preoperative diagnosis indicates whether laparoscopic myomectomy is possible or whether laparotomy should be performed for large or numerous myomas. Each approach has its own indications.

The results of our study and many international series demonstrate the feasibility of laparoscopic and hysteroscopic myomectomy as a technique leading to remission of symptoms and a low rate of complications. The difficulty of the operation lies in achieving satisfactory haemostasis and an appropriate usage of sutures. The hysteroscopic myomectomy requires an operative hysteroscope and a well-trained gynaecological surgeon to use it [30]. Severe haemorrhages can be tackled and adhesions can be prevented.

## Figures and Tables

**Table 1:** Baseline characteristics of the study participants.

	Group I	Group II	*P* value
	195 patients	140 patients	
Age, years (mean ± SD)	34.9 ± 2.9 (range, 26–48)	35.6 ± 3.8 (range, 23–50)	*P* > 0.05
BMI (mean ± SD)	21.8 ± 1.7	22.9 ± 1.3	*P* > 0.05
Parity (mean ± SD)	1.2 ± 0.3	1.3 ± 0.9	*P* > 0.05

**Table 2:** Myoma and surgical procedure outcome of the study participants.

	Group I	Group II	*P* value
	195 patients	140 patients	
Infiltration of myometrium (no. of patients)	18 (17.6%)	10 (14.9%)	>0.05
Total operative laparoscopic time (minutes)	60 ± 7.2 (53–67)	97 ± 8.9 (89–105)	<0.05
Intrasurgical blood loss (mL)	140 ± 4.7 (135–145)	175 ± 6.8 (168–182)	>0.05
Catheter inside pelvis for postsurgical drainage (no. of patients)	78 (40%)	51 (36.4%)	>0.05
Need for painkiller drugs (no. of patients)	81 (41.5%)	56 (40%)	>0.05
Fever (no. of patients with fever > 38°C after 24 hours and for the first 2 days of hospitalization)	22 (11.2%)	13 (9.2%)	>0.05
Therapeutic postoperative antibiotics administration (no. of patients)	16 (8.2%)	9 (6.4%)	>0.05
Duration of hospital stay: discharging in 24 hours (no. of patients)	140 (71.7%)	99 (70.7%)	>0.05
Duration of hospital stay: discharging in 48 hours (no. of patients)	55 (28.2%)	41 (29.2%)	>0.05
US hematoma (>3 cm) detected in myometrium (no. of patients)	13 (6.6%)	8 (5.7%)	>0.05

Data are presented as mean ± standard deviation or median range.

**Table 3:** Short-term complications after laparoscopic intracapsular myomectomy.

	Group I	Group II	*P* value
	195 patients	140 patients	
Hemoglobin preoperative (range in g/dL)	11.8 ± 2.8	12.1 ± 1.6	>0.05
Hemoglobin postoperative (range in g/dL)	10.4 ± 1.7	11.2 ± 1.9	>0.05
Hematocrit preoperative (range in %)	36.3 ± 3.9	36.8 ± 4.7	>0.05
Hematocrit postoperative (range in %)	33.2 ± 5.6	32.9 ± 8.1	>0.05
Red cells preoperative (range in milions/mm^3^)	4209 ± 227.2	4324 ± 235.1	>0.05
Red cells postoperative (range in milions/mm^3^)	4120 ± 193.4	3990 ± 235.3	>0.05
Postoperative blood collection in the catheter inside pelvis (range in mL)	41 ± 5.3	38 ± 9.3	>0.05
Postoperative bladder pain after foley removal (no. of patients)	10 ± 6.4	9 ± 5.2	>0.05
